# Dynamic Alterations of miR-34c Expression in the Hypothalamus of Male Rats after Early Adolescent Traumatic Stress

**DOI:** 10.1155/2016/5249893

**Published:** 2016-01-26

**Authors:** Chuting Li, Yuan Liu, Dexiang Liu, Hong Jiang, Fang Pan

**Affiliations:** Department of Medical Psychology, Shandong University School of Medicine, No. 44 Wenhua Xi Road, Jinan, Shandong 250012, China

## Abstract

Several types of microRNA (miRNA) overexpression in the brain are associated with stress. One of the targets of miR-34c is the stress-related corticotrophin releasing factor receptor 1 mRNA (CRFR1 mRNA). Here we will probe into the short-term effect and long-term effect of early adolescent traumatic stress on the expression of miR-34c and CRFR1 mRNA. Traumatic stress was established by electric foot shock for six consecutive days using 28-day rats. The anxiety-like behaviors, memory damage, CRFR1 protein, CRFR1 mRNA, and miR-34c expression were detected in our study. The results of our study proved that exposure to acute traumatic stress in early adolescent can cause permanent changes in neural network, resulting in dysregulation of CRFR1 expression and CRFR1 mRNA and miR-34c expression in hypothalamus, anxiety-like behavior, and memory impairment, suggesting that the miR-34c expression in hypothalamus may be an important factor involved in susceptibility to PTSD.

## 1. Introduction

Posttraumatic stress disorder (PTSD) is a prevalent anxiety disorder triggered by the traumatic experiences which produce strong negative feelings, such as horror, intense fear, and helplessness [1]. The hypothalamic-pituitary-adrenal (HPA) axis plays a pivotal role in stress induced neural plasticity, so that dysregulation of HPA axis is responsible for susceptibility to certain anxiety disorders [2]. Further, as the key upstream factors in HPA axis, CRHR1 was considered as a critical factor in etiology and vulnerability of PTSD [3]. One study has showed that CRHR1 could strengthen the traumatic memories in limbic system of mice after exposure to foot shock stress [3]. Our previous study also showed that traumatic stress in early adolescence triggered long-term effect on central CRFR1 expression and induced dysfunction of HPA axis in adulthood [4].

MicroRNAs (miRNAs), a subset of endogenous small RNA molecules, are widely expressed in astrocytes and neurons in the brain and perform crucial regulatory functions in gene expression in the central nerve system [5, 6]. Previous studies have found that dicer1 and miR-17 expression were increased in reactive astrocyte, and it is also reported that dicer1 plays an important role in astrocyte development [7]. miRNAs exert their function via base-pairing with complementary sequences within mRNA molecules. Upon sequence-specific binding of miRNAs, mRNA molecules are destabilized through shortening of their poly(A) tails or degraded by cleavage of the mRNA strand or less efficiently translated into proteins by ribosomes [8, 9]. One study has observed that miRNAs are expressed differentially in patients with different psychiatric diseases. Meanwhile, stress, glucocorticoids, and mood stabilizers altered the miRNAs level of the patients, suggesting that miRNAs may be the potential vital factors of the pathophysiology and therapeutics of mental diseases [10]. More studies also pointed out that certain miRNAs may act as epigenetic modulators of gene expression in psychiatric disorders like autism, schizophrenia, major depression, and anxiety [10–12]; specific miRNAs were related to neuronal differentiation and synaptic plasticity and the treatment target of anxiety disorders [13]. Those studies indicated that the alterations of certain miRNAs expression had implication in pathogenesis of PTSD [10, 14].

miR-34c is a stress-related miRNA which was prominently increased after traumatic stress and associated with decreased anxiety-like behaviors. What caught our attention was that CRFR1 mRNA is the target of miR-34c. Previous work has confirmed that miR-34c combined with an evolutionarily conserved region in the 3′UTR of CRFR1 mRNA perform its effect. miR-34c reduces the expression of stress-related proteins (such as CRFR1) and plays a role in the recovery process of stress reaction, suggesting that it might have vital implication in vulnerability to PTSD and might become a new target for the prevention and treatment of stress-related disorders [15].

Adolescence is a very rapid development period, which had increased susceptibility to stress. Recent studies have showed that early life stress may have more influence on epigenetic states and brain function than similar stress exposure later in life [16]. Our previous study showed that early life stress increased susceptibility to stress through CRFR1 expression in brain [4]; another study has observed that early life stress activated REST4-mediated gene transcription in the medial prefrontal cortex [17]. Those studies provided new insights that miRNAs could regulate gene expression which alters susceptibility to developing stress-related diseases in adulthood after early life stress. However, miR-34c expression and the association between miR-34c and CRF1 expression in hypothalamus in adult rats after adolescent stress had not clarified.

In the present study, we used our previously established rat model for PTSD, which replicates the specific neuroendocrinological abnormalities observed in PTSD patients [4, 18]. We would observe miR-34c expression in the hypothalamus after adolescent stress because this brain region is a complex region considered to be part of the limbic system and integrate the nervous system and the endocrine system and act as a “switching station” in the brain [19]. The purpose of the study was first to detect dynamic changes, including short-term state and long-term state of miR-34c expression after early adolescent exposure to the stress. Second, we probe into the question whether miR-34c expression could timely regulate CRFR1 expression by using CRFR1 antagonist to block the CRFR1 activity.

## 2. Methods and Materials

### 2.1. Animals

**Figure 1:**
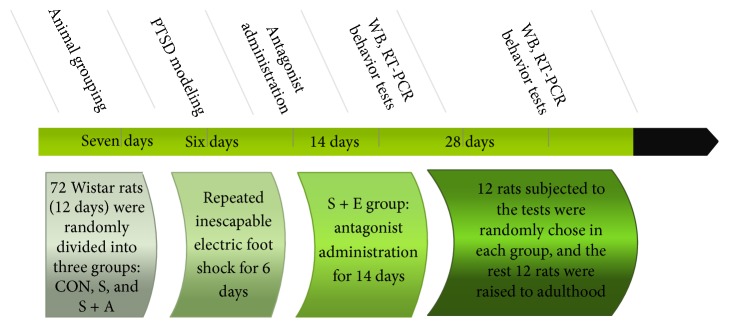


A total of 72 male Wistar rats (21 days old, obtained from the experimental animal center of Shandong University, China) were group-caged (two or three per cage) under controlled lighting conditions (07:00–19:00 h) and temperatures (25 ± 2°C) with food and water made available ad libitum and allowed to acclimate for seven days prior to experimental testing. The study was approved by the Institutional Animal Care Committee of Shandong University. Rats were randomly divided into three groups (*n* = 24 in each group): the control group (CON), the stress group (S), and the stress and antagonist group (S + A). After animal modeling and drug administration (two weeks after foot shock), 12 rats randomly chosen from each group were sacrificed after behavioral tests. The rest of the animals were raised to adulthood (six weeks after foot shock) and were sacrificed after behavioral tests (see Figure 1).

### 2.2. Animal Model of PTSD

With the exception of the control group, the rats received the repeated inescapable electric foot shock for six consecutive days, according to the previously published method [4, 18]. In each day, there were two trials which lasted for 30 minutes; the interval between the two trials was not less than 4 hours. In each trial, electric foot shock continued for 6 seconds and repeated 20 times with a random interval. The current intensity of electric foot shock was 0.5 mA.

### 2.3. Antagonist Administration

CRFR1 antagonist CP-154, 526 (Sigma-Aldrich, USA) was administered intraperitoneally. The rats in S + A group were treated with CP-154, 526 (3.2 mg/kg/day, in vehicle) for 14 days after the foot shock stress. Rats in other groups were treated with vehicle (80% polyethylene glycol 400) to balance the systematic error. The dose of CP-154, 526 was determined according to a previous study [20].

### 2.4. Elevated Plus Maze (EPM)

The elevated plus maze consisted of three parts and two opposite closed arms (50 × 10 cm^2^) with 40 cm tall nontransparent walls, two opposite open arms (50 × 10 cm^2^), and a central part (10 × 10 cm^2^), which was elevated 50 cm above the floor. The laboratory room was maintained with controlled levels of light and temperatures. The rats were individually placed in the center part of the maze facing an open arm and allowed free exploration for 5 minutes. The apparatus was completely cleaned with 75% ethanol between two sessions. The number of entries into each arm and the total time spent in each arm were recorded by the SMART video tracking system (SMART v3.0, Panlab, Spain). Ratio entry was defined as the total entries into the open arms divided by the total entries into any arm of the maze. Ratio time was defined as the total time spent in the open arms divided by the total time spent in any arm of the maze. Anxiety score was calculated as anxiety score = 1 − (ratio time + ratio entry/2). Anxious rats were more likely to stay in the closed arms so that a reduced ratio entry or ratio time indicates a more anxious status. When the ratio entry and ratio time are zero, the anxiety score is 1, which means extreme anxiety [21].

### 2.5. Morris Water Maze

The test was carried out within 24 h after EPM test. The water maze was a cylindrical black galvanized metal container that was 120 cm in diameter and equipped with a platform 1-2 cm below the water surface. The visual objects were placed at fixed positions to serve as visual cues for the location of the platform. The swimming track of the animals in the water maze was recorded and measured by the SMART video tracking system (SMART v3.0, Panlab, Spain). At the start of learning trail, animals were placed on the platform for 10 s to familiarize themselves with the environment. Then the animals were individually placed in the water facing the wall of the water maze and trained to find the platform from different locations (E, S, W, and N) around the edge of the container for 5 consecutive days. Once the animals reach the platform, the trial was terminated. If the animals failed to find the platform within 60 s, the animal was placed on the platform for 10 s and the latency was recorded as 60 s. The time(s) of escape latency to find the platform was recorded and measured by the SMART video tracking system (SMART v3.0, Panlab, Spain). On day 6, the original platform was removed and this quadrant was defined as target quadrant. The animals were placed in the quadrant opposite the platform and allowed free exploration for 1 min. The entries to the target quadrant and cumulative time spending in the target quadrant were recorded and measured by the SMART video tracking system (SMART v3.0, Panlab, Spain).

### 2.6. Western Blotting

#### 2.6.1. Tissue Preparation

Six rats in each group were decapitated immediately after behavioral tests. The rat's skull was cut and both sides of the frontal and the parietal bone were pulled off to collect the whole brain from the cranial cavity. After that, the hypothalamus was collected and immersed immediately in liquid nitrogen and stored at −80°C for further protein isolation.

#### 2.6.2. Protein Isolation

To isolate protein sample, the brain tissue was homogenized in the lysis buffer (50 mM Tris (pH 7.4), 150 mM NaCl, 1% Triton X-100, 1% sodium deoxycholate, 0.1% SDS, sodium orthovanadate, sodium fluoride, EDTA, and leupeptin) supplemented with 1% protease inhibitor phenylmethanesulfonyl fluoride (PMSF) in a ratio of 1 : 5 (1 g tissue/5 mL reagent). The lysed tissue sample was centrifuged at 14000 g at 4°C for 30 min, and then the protein-containing supernatant obtained was either immediately used or stored at −80°C. The protein concentration was detected by BCA Protein Assay Kit (Beyotime Institute of Biotechnology) using the iMark Microplate Absorbance Reader (Bio-Rad, CA, USA).

#### 2.6.3. CRFR1 Western Blotting

Brain protein samples containing the same amount of total proteins were mixed with a 6x Laemmli loading buffer (Tris-HCl, 50 mM, pH 6.8; dithiothreitol, 0.1 M, pH 6.8; glycerol, 10%; sodium dodecyl sulfate (SDS), 2%; and bromophenol blue, 0.02 mg/mL). The mixed protein sample was heated at 99°C for 5 min to cause protein denaturation, and then 20 *μ*g of protein sample was separated on 12% sodium dodecyl sulfate-polyacrylamide (SDS-PAGE) gel and electrotransferred to polyvinylidene difluoride (PVDF) membranes (Bio-Rad, CA, USA). The membrane was blocked with 5% BSA in TBS containing 0.1% tween-20 (TBST) for 1 h and incubated with primary antibodies against CRFR1 (1 : 4000, Sigma-Aldrich, USA, SAB4500465) or GAPDH (1 : 4000, Biogot Technology, Co., Ltd.) at 4°C overnight in a refrigerator. On the following day, after washing with TBST for 5 min three times, the PVDF membrane was incubated for 1 h at room temperature with the horseradish peroxidase- (HRP-) conjugated secondary antibody (1 : 10000). Then, the PVDF membrane was washed again with TBST for 15 min three times, the Western blots were visualized after being incubated with ECL solution (Millipore Corp., Billerica, Massachusetts, USA) for 1 min and exposed onto photographic films (Eastman Kodak Company, Rochester, New York, USA) for 10–90 sec. Signal intensities were quantified by the Image J 14.0 software and the density value of the objective protein band was normalized according to that of the GAPDH band of the same sample.

### 2.7. Real-Time PCR Assay

Six rats in each group were used to detected CRFR1 mRNA and miR-34c expression. Each hypothalamus was mixed with 1 mL Trizol (Invitrogen) to extract total RNA from frozen samples. 1 mL of RNA was used to measure the expression of CRFR1 mRNA or miR-34c by RT-PCR. The expression of GAPDH or U6 was used as internal control. The expression of CRFR1 mRNA or miR-34c was calculated according to the threshold cycle (Ct); the CT of the target gene for each sample was corrected by subtracting the CT of the internal control (ΔCT). The controls were chosen as reference samples with mean ΔCT for the control samples being subtracted from the ΔCT for all the experimental samples (ΔΔCT). Finally, relative expression levels were calculated as 2^−[(Ct  of  CRFR1  mRNA)−(Ct  of  GAPDH)]^ or 2^−[(Ct  of  miR-34c)−(Ct  of  U6)]^. Real-time PCR experiments were performed by Kangchen Bio-Tech, Shanghai, China.

### 2.8. Statistical Analysis

All analyses were carried out using the statistical software SPSS18.0. The repeated-measures analysis of variance (ANOVA) was used for the analysis of the escape latency among different groups in the Morris water maze. One-way ANOVA was used for the analysis of the other dates. Post hoc analyses consisted of *F* tests for simple effects and Turkey's and Games-Howell tests where appropriate. Results were expressed as the mean ± standard error of the mean (SEM). Significances were accepted to be present at *p* < 0.05.

## 3. Results

### 3.1. Behavioral Test

#### 3.1.1. EPM Test

**Figure 2:**
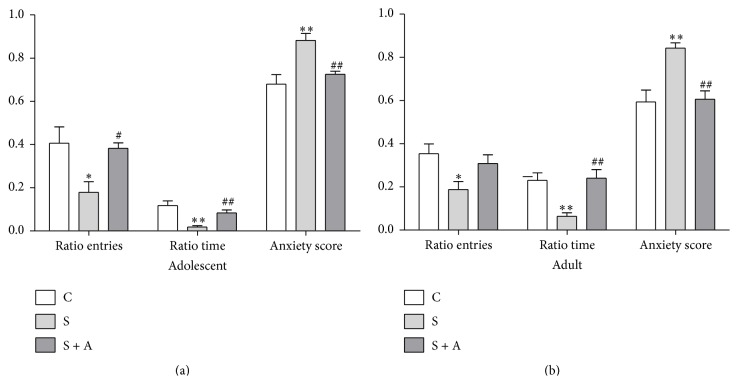
Anxiety-like behavior in the EPM test (*n* = 8 in each group). (a) Anxiety-like behavior of rats in the EPM test in adolescence. (b) Anxiety-like behavior of rats in the EPM test in adulthood. Ratio entry was defined as the total entries into the open arms divided by the total entries into any arm of the maze. Ratio time was defined as the total time spent in the open arms divided by the total time spent in any arm of the maze. Anxiety score was calculated as anxiety score = 1 − (ratio time + ratio entry/2). Values were expressed as mean ± SEM. *∗* and *∗∗* indicate *p* < 0.05 and *p* < 0.01 versus CON, respectively; # and ## indicate *p* < 0.05 and *p* < 0.01 versus S, respectively.

Figure 2(a) shows the ratio entry to the open arm [*F*(2, 22) = 5.444, *p* = 0.012], ratio time in the open arm [*F*(2, 22) = 10.914, *p* = 0.001], and anxiety score [*F*(2, 22) = 10.575, *p* = 0.001] displayed by the rats two weeks after foot shook. S group had lower ratio entry than the CON (*p* = 0.018) and S + A (*p* = 0.035) groups. Ratio time in the open arm was lower in the S group than the CON (*p* = 0.005) and S + A (*p* = 0.005) groups. Anxiety scores were higher in the S group than the CON (*p* = 0.007) and S + A (*p* = 0.003) groups.  Figure 2(b) shows the long-lasting effects of adolescent foot shock on anxiety-like behaviors of rats later in adulthood. Similar group differences were observed in ratio entry to the open arm [*F*(2, 22) = 4.32, *p* = 0.027], ration time in the open arm [*F*(2, 22) = 9.495, *p* = 0.001], and anxiety score [*F*(2, 22) = 11.473, *p* = 0.000]. S group had lower ratio entry than the CON group (*p* = 0.025), lower ratio time than the CON (*p* = 0.004) and S + A (*p* = 0.002) groups, and higher anxiety score than the CON (*p* = 0.001) and S + A (*p* = 0.002) groups.

#### 3.1.2. Memory Function Test

**Figure 3:**
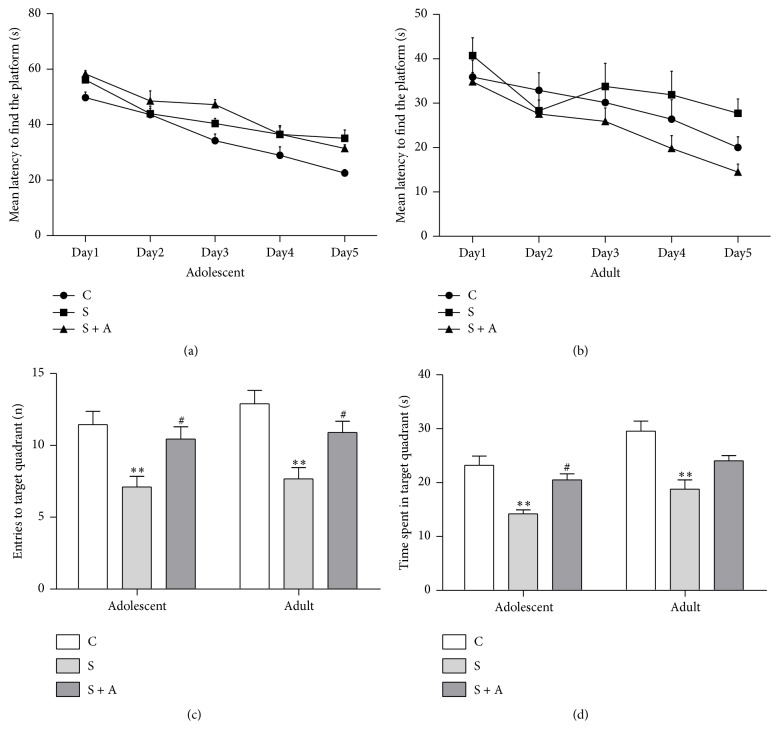
Spatial memory performance in the Morris water maze (*n* = 8 in each group). (a) Mean escape latency to the platform in the Morris water maze in adolescence. (b) Mean escape latency to the platform in the Morris water maze in adulthood. (c) Entries to the target quadrant in adolescence and adulthood. (d) Time spent in the target quadrant in adolescence and adulthood. Values were expressed as mean ± SEM. *∗∗* indicates *p* < 0.01 versus CON; # indicates *p* < 0.05 versus S.

The Morris water maze was performed two weeks and six weeks after foot shock. The mean time to find the platform in the training days is shown in Figures 3(a) and 3(b), and repeated-measures ANOVA confirmed that there was no obvious difference among these groups. One-way ANOVA showed similar differences in the number of entries (Figure 3(c)) and the time spent in the target quadrants (Figure 3(d)) of the adolescent rats and adult rats. Two weeks after foot shock, S group performed less entries to the target quadrants than CON (*p* = 0.004) and S + A (*p* = 0.026) groups [*F*(2, 22) = 7.252, *p* = 0.003] and lower time spent in the target quadrants than CON (*p* = 0.004) and S + A (*p* = 0.026) groups [*F*(2, 22) = 13.482, *p* = 0.000]. Six weeks after foot shock, S group performed less entries to the target quadrants than CON (*p* = 0.001) and S + A (*p* = 0.031) groups [*F*(2, 22) = 9.88, *p* = 0.001] and lower time spent in the target quadrants than CON (*p* = 0.000) group [*F*(2, 22) = 11.883, *p* = 0.000].

### 3.2. CRFR1 Expression in Hypothalamus

**Figure 4:**
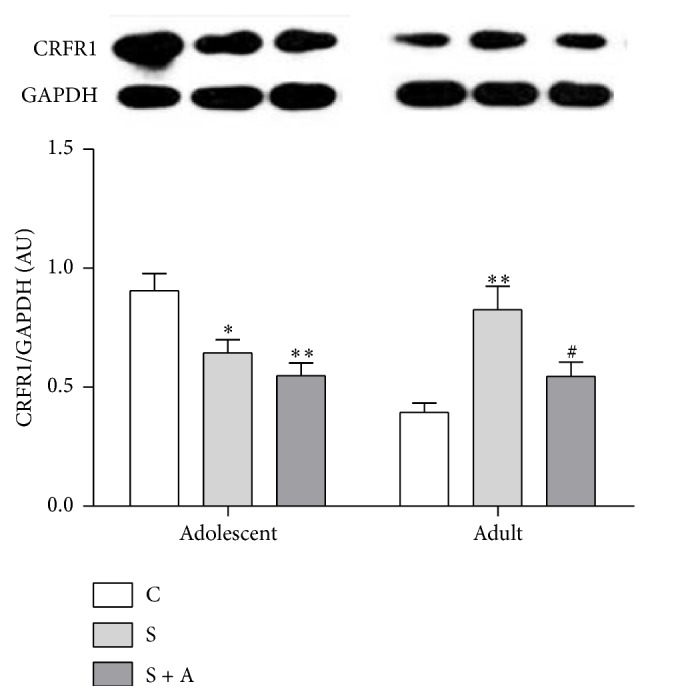
Representative images of Western blotting for CRFR1 expression in adolescent and adult hypothalamus. Values were expressed as mean ± SEM. *∗* and *∗∗* indicate *p* < 0.05 and *p* < 0.01 versus CON, respectively; # indicates *p* < 0.05 versus S.

In the adolescent hypothalamus [*F*(2, 16) = 9.275, *p* = 0.002; see Figure 4], the S group (*p* = 0.021) and S + A group (*p* = 0.002) had lower CRFR1 expression than the CON group. However, in the adult hypothalamus [*F*(2, 16) = 9.706, *p* = 0.002; see Figure 4], S group exhibited higher CRFR1 expressions than the CON group (*p* = 0.002) and S + A group (*p* = 0.032).

### 3.3. CRFR1 mRNA Expression and miR-34c Expression in Hypothalamus

**Figure 5:**
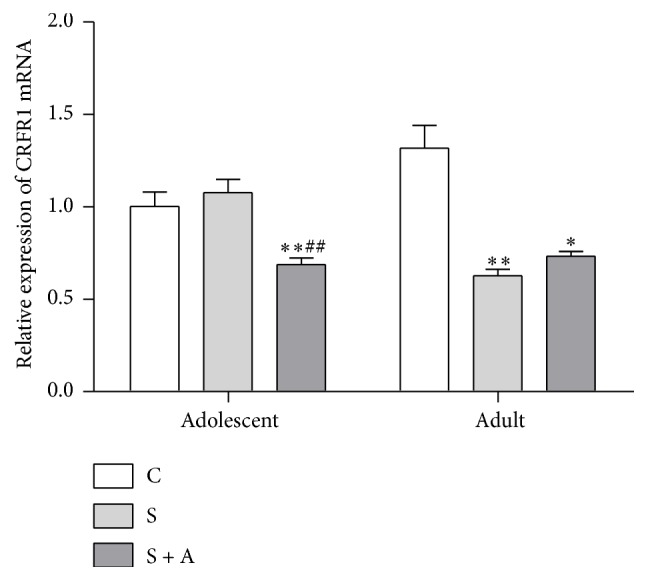
Relative expression of CRFR1 mRNA in adolescent and adult hypothalamus. Values were expressed as mean ± SEM. *∗* and *∗∗* indicate *p* < 0.05 and *p* < 0.01 versus CON, respectively; ## indicates *p* < 0.01 versus S.

The level of CRFR1 mRNA was lower in the adolescent hypothalamus in the S + A group than CON (*p* = 0.009) and S groups (*p* = 0.002) [*F*(2, 16) = 10.493, *p* = 0.001; see Figure 5]. In the adult hypothalamus [*F*(2, 16) = 24.650, *p* = 0.000; see Figure 5], the S (*p* = 0.004) and S + A (*p* = 0.01) groups had lower level of CRFR1 mRNA than the CON group.

**Figure 6:**
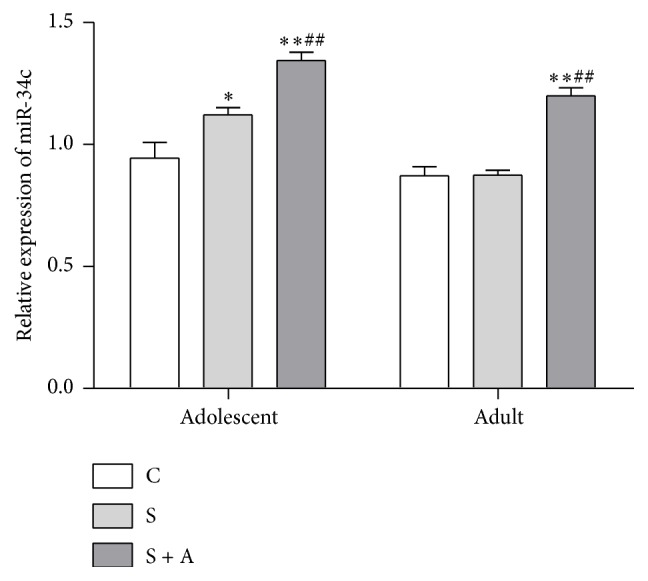
Relative expression of miR-34c in adolescent and adult hypothalamus. Values were expressed as mean ± SEM. *∗* and *∗∗* indicate *p* < 0.05 and *p* < 0.01 versus CON, respectively; # indicates *p* < 0.05 versus S.

In the adolescent hypothalamus [*F*(2, 16) = 9.272, *p* = 0.002; see Figure 6], the S group (*p* = 0.011) and the S + A group (*p* = 0.003) had higher miR-34c expression than the CON group. However, in the adult hypothalamus [*F*(2, 16) = 8.547, *p* = 0.003; see Figure 6], the S + A group exhibited higher miR-34c expressions than the CON group (*p* = 0.005) and S group (*p* = 0.012).

## 4. Discussion

Early life adverse conditions may lead to abnormal behavioral, neuroendocrine, and genetic responses which might be involved in the pathogenesis of psychiatric disorders [22–27]. Stressful experiences and individual psychology hereditary quality are recognized as risk factors for PTSD [28, 29]. In this study, we focused on the short-term and long-term effects of adolescent foot shock on anxiety-like behavior, memory damage, protein CRFR1 expression, CRFR1 mRNA, and miR-34c levels in the hypothalamus of male Wistar rats.

Behavioral tests were carried out two weeks and six weeks after the stressful foot shock, which acts as models of short-term and long-term effects of adolescent stress, like what to be observed in the PTSD patients. We found that foot shock had both short-term and prolonged negative effects on anxiety-like behavior and memory. Gratifyingly, CRFR1 antagonist performed positive effects. Adolescent foot shock triggered more anxiety-like behaviors and reduced open arm exploration in the EPM test, and passing time did not erase the anxiety of the stressed rats. Since the stressed rats performed fewer entries to the target quadrant and spent less time there than the controls, we speculated that rats exposed to foot shock suffered memory damage which lasted to adulthood. Administration of CRFR1 antagonist CP-154,526 after foot shock was proved useful for alleviation of anxiety and memory damage immediately and persistently. Our results supported the conclusions that early life stress resulted in a persisted anxiety behavior and PTSD patients or animal models may develop memory impairments [30–32], which are also affected by individual characters and the type and intensity of stress [26, 30].

Our previous study found that early adolescent stress led to lasting and profound changes in central CRFR1 expression [4]. Therefore, we want to reveal whether the changes of CRFR1 expression occur with the accompanying changes in CRFR1 mRNA expression and miR-34c expression in the hypothalamus, as one of the miR-34c targets is the CRFR1 mRNA, which was regulated via the complementary site on its 3′UTR [15]. In adolescent study, stressed rats showed similar level of CRFR1 mRNA, increased miR-34c expression, and decreased CRFR1 expression compared with the controls in hypothalamus. These results supported the idea that traumatic stress could induce increased miR-34c and decreased CRFR1 expression, which is consistent with the mechanism about miRNA influencing the protein translation [8]. As a marker in stress recovery process, higher level of miR-34c was observed in S + A group. Combined with the result of improved behaviors in CRFR1 antagonist group, our study suggested that CRFR1 antagonist could target a positive process including increased level of miR-34c during acute stress reaction and give a new certification that miR-34c might be closely related with vulnerability to PTSD.

The study focused on the relationship among levels of CRFR1, CRFR1 mRNA, and miR-34c expression in adult stressed rats. In consistent with our hypothesis, stressed rats showed lower level of CRFR1 mRNA, similar level of miR-34c, and increased CRFR1 expression compared with the unstressed rats. This means although the behavioral performance is the same between adolescence and adulthood of rats after adolescent stress, the CRFR1 mRNA, miR-34c, and protein CRFR1 displayed different dynamic changes after certain period of passing time. The reasons may be explained partially with the homeostasis theory of stress: when individual confronts stress stimulus, organisms would start self-defense mechanism to cope with the stress and induced physiological change to maintain homeostasis. For instance, miR-34c was upregulated after exposure to acute stress, performing anxiolytic properties [15]. In our study, after adolescent foot shock, stressed rats showed increased level of miR-34c in the short term which trigged lower expression of CRFR1 in the hypothalamus to fight against the anxiety. After a protracted struggle which did not work, the level of miR-34c expression returned to normal gradually, while the level of CRFR1 expression was upregulated, as we observed in the hypothalamus six weeks after foot shock. In addition, the sustained upregulated CRFR1 expression might trigger a negative feedback action on the CRFR1 mRNA to maintain the homeostasis. Both positive and negative stress adaptations induce the experience epigenetic changes that affect its future responses [33]. The other reasons including the epigenetic factors such as DNA methylation, histone acetylation, and other types of noncoding RNAs [16, 34, 35] might also influence the CRFR1 expression. For instance, Elliott et al. revealed CRH promoter was demethylated in stress-vulnerability mice while imipramine treatment could reverse the alterations of CRH promoter methylation, mRNA expression, and behavior [36]. It is noticed that there were prominent higher levels of miR-34c in both adolescent and adult CRFR1 antagonist group in the study. The results indicated CRF1 antagonist might improve anxiety-like behavior and memory by alteration of miR-34c expression in hypothalamus.

## 5. Conclusions

Our results demonstrated that severe traumatic stress in early adolescent induced lasting effects on anxiety-like behavior and spatial memory damage, different alterations of CRFR1 expression, and CRFR1 mRNA and miR-34c expression in hypothalamus between adolescent and adult period, which suggested that the miR-34c expression in hypothalamus may be unique regulator of stress reaction and may play a role in vulnerability to PTSD following exposure to traumatic experience.

## Abbreviations

ANOVA: Analysis of variance
CRF: Corticotrophin releasing factor
CRFR1: Corticotrophin releasing factor receptor 1
EPM: Elevated plus maze
HPA: Hypothalamic-pituitary-adrenal
PFC: Prefrontal cortex
PTSD: Posttraumatic stress disorder
PVDF: Polyvinylidene difluoride
SEM: Standard error of the mean
SDS-PAGE: Sodium dodecyl sulfate-polyacrylamide.
